# Prediction of Subsurface Fatigue Damage in Dental CAD/CAM Restorations: Intraoral Scanning vs. Optical Coherence Tomography

**DOI:** 10.3390/bioengineering13070808

**Published:** 2026-07-14

**Authors:** Christoph Moos, Julie-Jacqueline Kuhl, Bernd Wöstmann, Christin Grill, Ralf Brinkmann, Maximiliane Amelie Schlenz

**Affiliations:** 1 Department of Prosthodontics, University Hospital Schleswig-Holstein, Christian Albrecht University of Kiel, Campus Kiel, Arnold-Heller-Str. 3, 24105 Kiel, Germany; maximiliane.schlenz-helmke@uksh.de; 2 Department of Prosthodontics, Justus Liebig University Giessen, Schlangenzahl 14, 35392 Giessen, Germany; bernd.woestmann@dentist.med.uni-giessen.de; 3 Medical Laser Center Lübeck, Peter-Monnik-Weg 4, 23562 Lübeck, Germanyralf.brinkmann@uni-luebeck.de (R.B.); 4 Institute of Biomedical Optics, University of Lübeck, Peter-Monnik-Weg 4, 23562 Lübeck, Germany

**Keywords:** CAD/CAM materials, dental restoration, diagnostic imaging, materials fatigue, optical coherence tomography, subsurface damage

## Abstract

This study extended a previously established intraoral scanning (IOS) and optical coherence tomography (OCT) dual-modality monitoring workflow for computer-aided design/computer-aided manufacturing (CAD/CAM) restorations to three additional crown material classes alongside a resin composite (RECO) reference. Four material classes were investigated (n=8 each): RECO, polymer-infiltrated ceramic network (PICN), lithium disilicate ceramic (LDSC), and zirconia-reinforced lithium silicate ceramic (ZLSC). Monolithic crowns were adhesively luted to standardized human molar abutment teeth and aged by cyclic loading (50 N to 500 N, 2 Hz, (37 ± 2) °C, up to 1,250,000 cycles) in a mouth-motion simulator. IOS and handheld OCT were performed at baseline and after every 250,000 cycles under phantom-head conditions; correspondence was assessed using Spearman’s rank correlation coefficient (exploratory, uncorrected for multiple comparisons). OCT consistently showed higher defect extents than IOS across all material classes and timepoints. While no significant IOS-OCT associations were found for RECO and the PICN, OCT detected full-thickness vertical subsurface damage propagation from the earliest timepoint in LDSC and ZLSC, with IOS-derived surface wear remaining markedly lower. Surface-based monitoring alone did not reliably reflect subsurface damage propagation, a dissociation most pronounced in the vertical dimension and silicate-based materials. Intraoral OCT may provide complementary, non-invasive subsurface information to support individualized recall scheduling and minimally invasive repair decisions.

## 1. Introduction

In contemporary digital workflows, longitudinal monitoring of computer-aided design/computer-aided manufacturing (CAD/CAM) restorations is primarily based on the superimposition of sequential intraoral scanning (IOS), which quantifies external surface wear but cannot detect damage beneath the restoration surface [1,2]. As a consequence, subsurface fatigue processes may progress undetected between recall appointments, highlighting the need for an adjunct imaging modality capable of detecting subsurface defects under clinically relevant conditions [1,3].

CAD/CAM technology allows restorations to be machined from prefabricated industrial blocks with high material homogeneity and standardized mechanical properties and—depending on the system—favorable machinability and the option of intraoral repair [1,4,5,6]. Monolithic posterior crowns can be fabricated from materials spanning a wide range of mechanical behavior, from comparatively compliant resin composites (RECOs) and polymer-infiltrated ceramic networks (PICNs) to high-strength glass-ceramics such as lithium disilicate (LDSC) and zirconia-reinforced lithium silicate (ZLSC), each characterized by distinct elastic moduli, strengths, and reliability profiles [3,7,8,9,10]. With the increase in ceramic content, elastic modulus and flexural strength increase, whereas the capacity for inelastic energy dissipation decreases, promoting a transition from damage-tolerant behavior to brittle fracture [7,8,11]. Because fatigue resistance is determined by material-specific mechanical properties, damage initiation and propagation mechanisms are expected to vary substantially among different material classes [3,12].

Failures of tooth-colored restorative materials are predominantly governed by fatigue damage resulting from repetitive subcritical loading rather than by single catastrophic overload events [13,14,15]. Under occlusal loading, fatigue damage typically initiates subsurface or at the periphery of the contact area and develops through crack initiation, subcritical crack growth, and eventual partial or complete fracture [12,13,15,16,17,18]. Brittle silicate-based ceramics are particularly susceptible to this failure mode as cracks may propagate rapidly once initiated [12,19]. Detecting such damage at an early, still sub-clinical stage—before it has progressed through the full restoration thickness—is therefore of considerable clinical interest.

Conventional methods for characterizing fatigue damage are unsuitable for repeated in situ monitoring. Sectioning followed by light or scanning electron microscopy is destructive and restricted to post-test analysis, while high-speed imaging captures surface events without resolving internal structural changes [3,18,20,21]. Clinical tools—visual–tactile inspection, radiography, and IOS-based surface analysis—do not reliably detect cracks within crowns; radiography additionally involves ionizing radiation and is unsuitable for frequent follow-up [1]. A non-invasive, depth-resolved method is therefore required to monitor subsurface damage propagation over time.

Optical coherence tomography (OCT) addresses this need. Based on low-coherence interferometry, OCT reconstructs cross-sectional images from backscattered light with micrometer-scale resolution, without requiring contact or ionizing radiation [22,23,24]. Originally developed for ophthalmology, OCT is now applied across numerous medical disciplines [25]. In dentistry, it has been used in various applications, such as detecting carious lesions, evaluating the marginal and internal adaptation of restorations, assessing crowns and veneers, monitoring polymerization processes, and enabling image acquisition directly within the oral cavity [26,27,28,29,30,31,32]. Early handheld OCT scanning devices have likewise been developed and tested in vivo for dental imaging, providing cross-sectional B-scans of enamel and dentin [33], although their straight-beam design limits intraoral access compared to the angled relay probe used here, which redirects the beam to enable volumetric imaging throughout the oral cavity [1]. These capabilities position OCT as a promising candidate for the non-invasive assessment of subsurface fatigue damage in dental restorations [3].

To date, evidence on OCT-based fatigue monitoring of CAD/CAM restorations remains limited. Schlenz et al. [3] monitored fatigue damage across several CAD/CAM material classes with OCT but used a bench-top system unsuitable for intraoral application and did not directly relate subsurface damage to external surface wear. Yazigi et al. [29] examined crack progression in lithium disilicate occlusal veneers with a laboratory OCT system and reported that pre-existing subsurface cracks were associated with more catastrophic failure, underlining the clinical relevance of subsurface damage assessment independent of surface appearance. More recently, Kuhl et al. [1] established a reproducible chairside dual-modality workflow combining routine IOS with a handheld intraoral OCT device and demonstrated, for a single CAD/CAM resin composite, that surface wear did not reliably reflect subsurface fatigue damage. Whether this dissociation between surface and subsurface behavior generalizes to other material classes—particularly to brittle silicate-based ceramics with fundamentally different fracture characteristics—has not yet been investigated.

The present study therefore extended the reproducible dual-modality chairside workflow established in Kuhl et al. [1]—combining routine IOS with a handheld intraoral dental OCT device—to three additional CAD/CAM crown material classes (PICN, LDSC, and ZLSC) alongside the previously investigated RECO as a reference. Fatigue damage was monitored at predefined load-cycle intervals under phantom-head conditions using IOS and handheld OCT, and agreement between the two modalities was quantified using matched vertical and horizontal metrics. The null hypothesis was that surface wear measured by IOS is not associated with subsurface damage propagation measured by OCT, regardless of restoration material.

## 2. Materials and Methods

### 2.1. Specimen Preparation

A total of n=8 specimens per restoration material were prepared following the protocol established in previous studies [1,3,34]. Briefly, caries-free human molars were collected with informed consent with approval of the local ethics committee (Ref. No. 143/09), disinfected, and stored hydrated in distilled water. Standardized abutment teeth were prepared by computer numerical control (CNC) milling (Mikron HSM 400, GF Machining Solutions GmbH, Schorndorf, Germany) to ensure identical geometry across all specimens. Monolithic posterior crowns were CAD/CAM-milled (CORiTEC 250i, imes-icore, Eiterfeld, Germany) from four different restorative materials representing distinct material classes: RECO (Brilliant Crios, Coltene, Altstätten, Switzerland), PICN (Vita Enamic, VITA Zahnfabrik, Bad Säckingen, Germany), LDSC (IPS e.max CAD, Ivoclar Vivadent, Schaan, Liechtenstein), and ZLSC (Cerec Tessera, Dentsply Sirona, Bensheim, Germany). Prior to cementation, LDSC crowns were crystallized without glaze (Programat EP3000, Ivoclar Vivadent, Schaan, Liechtenstein; Programme 1), and ZLSC crowns received a glaze firing (Programat EP3000; CEREC Universal Spray Glaze Fluo, Dentsply Sirona, Bensheim, Germany) according to the manufacturer’s instructions.

Crowns were polished, conditioned, and adhesively luted to the prepared abutment teeth according to the respective manufacturer’s instructions using material-specific luting systems. Surface pre-treatment differed by material class: RECO crowns were air-abraded prior to bonding, whereas all other material classes (PICN, LDSC, and ZLSC) were conditioned with hydrofluoric acid and silanized. All crowns were luted with material-specific dual-cure resin cements under a standardized seating force and light-polymerized with a calibrated polywave LED curing unit (Bluephase G2, Ivoclar Vivadent, Schaan, Liechtenstein; ≥800 mW/cm^2^). Detailed pre-treatment parameters and luting protocols are summarized in Table 1. All specimens were stored in distilled water at (37 ± 2) °C for ≥24 h prior to testing and maintained in a hydrated state throughout.

### 2.2. Artificial Aging, Monitoring, and Outcome Measures

Artificial aging, dual-modality monitoring, and outcome measurement followed the protocol described in detail in Kuhl et al. [1]; only a brief summary is provided here. Specimens were aged in a computer-controlled mouth-motion simulator (prematecF1000, wl-tec, Wertheim, Germany) under vertical cyclic loading between 50 N and 500 N at 2 Hz in distilled water at (37 ± 2) °C, as the study prioritized a standardized IOS vs. OCT method comparison over full oral aging simulation. Loading was applied via single-point contact in the central fossa using a rounded stainless-steel antagonist (tip radius r=1 mm), representing a worst-case fatigue scenario. A total of 1,250,000 load cycles were applied, corresponding to approximately five years of clinical function [35].

**Figure 1:**
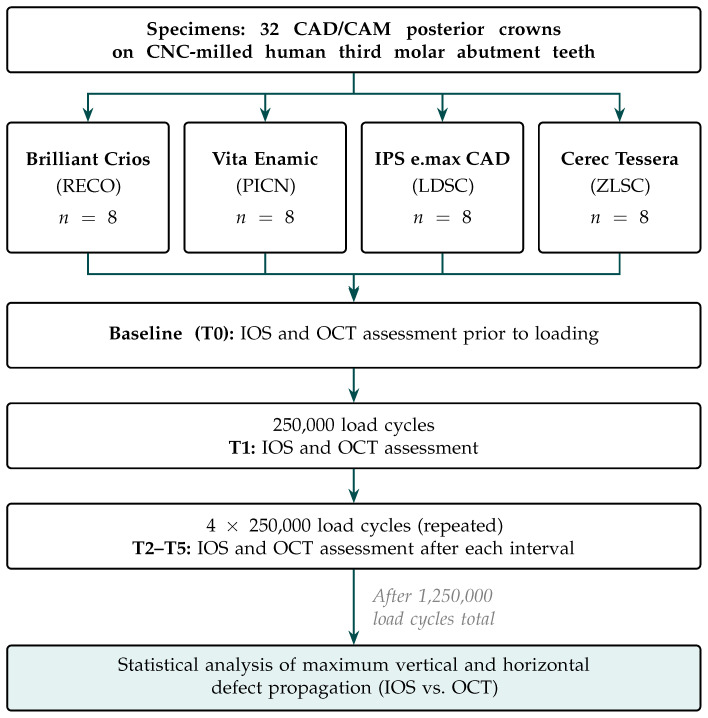
Schematic overview of the study protocol. CNC = computer numerical control; CAD/CAM = computer-aided design/computer-aided manufacturing; RECO = resin composite; PICN = polymer-infiltrated ceramic network; LDSC = lithium disilicate ceramic; ZLSC = zirconia-reinforced lithium silicate ceramic; IOS = intraoral scanning; OCT = optical coherence tomography.

IOS and OCT monitoring was performed at baseline (T0), and after 250,000 (T1), 500,000 (T2), 750,000 (T3), 1,000,000 (T4), and 1,250,000 (T5) load cycles (Figure 1), with each 250,000 cycles corresponding to approximately one year of intraoral function under average chewing conditions [35]. All assessments were conducted by a single calibrated examiner under clinical-close conditions in a dental phantom head with a model composed of human teeth; between simulation runs and imaging, specimens were kept continuously moist in distilled water. The same examiner performed the mechanical loading and all IOS and OCT acquisitions and analyses with knowledge of specimen material and timepoint; assessments were therefore not blinded, and blinding to timepoint was not feasible as progressive damage reveals the measurement sequence. Specimens that fractured prior to or at a given timepoint were assigned a maximum damage value of 100% for both IOS- and OCT-derived metrics, with this value retained for all subsequent timepoints to represent complete restoration failure.

IOS (Primescan AC, Dentsply Sirona, Bensheim, Germany) quantified external surface wear by superimposition of follow-up scans onto the T0 reference by best-fit alignment, analyzed in 3D analysis software (GOM Inspect Pro 2019, Carl Zeiss GOM Metrology, Braunschweig, Germany). Quadrant scans followed a standardized scanning path (occlusal to oral to buccal surfaces until full surface coverage was achieved) applied identically across all specimens and timepoints [2]. The maximum vertical and horizontal surface wear extents within the occlusal contact area between the T0 reference and each follow-up scan (T1–T5) were normalized to the local occlusal thickness (1.5 mm) and cusp-tip distance (7 mm), respectively.

Subsurface fatigue damage was assessed non-invasively using a custom handheld intraoral OCT device (optomechanical relay probe) coupled to a swept-source OCT system (Vega VEG210C1, Thorlabs, Newton, NJ, USA). A 25 μL distilled-water droplet was applied to the occlusal surface prior to each acquisition to reduce specular reflection and improve image contrast. Maximum vertical and horizontal defect extents were recorded from OCT volumes at each timepoint using ThorImage OCT (version 5, Thorlabs, Newton, NJ, USA) and normalized to local occlusal thickness and cusp-tip distance, respectively; subsurface damage is therefore reported as relative percentages rather than absolute metric values. Reproducible specimen positioning across timepoints was ensured by congruent basal alignment marks on the specimen holder and a dedicated insertion key.

### 2.3. Statistical Analysis

Descriptive statistics were calculated for IOS- and OCT-derived vertical and horizontal metrics at each of the five assessment timepoints (T1–T5) and are presented as mean ± standard deviation and median (interquartile range (IQR)). No a priori sample size calculation was conducted given the exploratory character of this multi-material investigation; instead, eight specimens per material class were tested, consistent with the sample size employed in the preceding single-material study and with specimen numbers commonly reported in comparable fatigue studies on CAD/CAM restorations [1,3,20,34,36]. To assess the correspondence between surface wear and subsurface damage, Spearman’s rank correlation coefficient (ρ) was computed between IOS- and OCT-derived metrics at each timepoint for each material individually, following the analytical approach established in Kuhl et al. [1]. To quantify the precision of each correlation estimate given the small sample size, 95% confidence intervals for ρ were calculated using a percentile bootstrap with 10,000 paired resamples (fixed random seed for reproducibility). Given the small sample size (n=8) and non-parametric data characteristics, a non-parametric approach was considered appropriate. All tests were two-sided with a significance level of α=0.05; *p*-values were not adjusted for multiple comparisons in keeping with the exploratory nature of the study. Statistical analyses were carried out in Python (version 3.12.3) using the SciPy library (version 1.15.1) [37].

## 3. Results

All RECO data shown in this section were taken from Kuhl et al. [1] and are included for cross-material comparison only.

### 3.1. Survival and Fatigue Damage

All RECO and LDSC specimens survived the complete artificial aging protocol without catastrophic failure (n=8 at all timepoints). One PICN specimen fractured between T0 and T1 and was excluded from further monitoring (n=7 at T1–T5). ZLSC showed the highest failure rate: three specimens fractured between T0 and T1, two additional specimens between T2 and T3, one between T3 and T4, and one between T4 and T5, leaving only a single specimen at T5 (n=5 at T1, n=5 at T2, n=3 at T3, n=2 at T4, and n=1 at T5). Fractured specimens were assigned a damage value of 100% for all subsequent timepoints. All catastrophic failure occurred within the material itself and was not caused by debonding.

**Figure 2:**
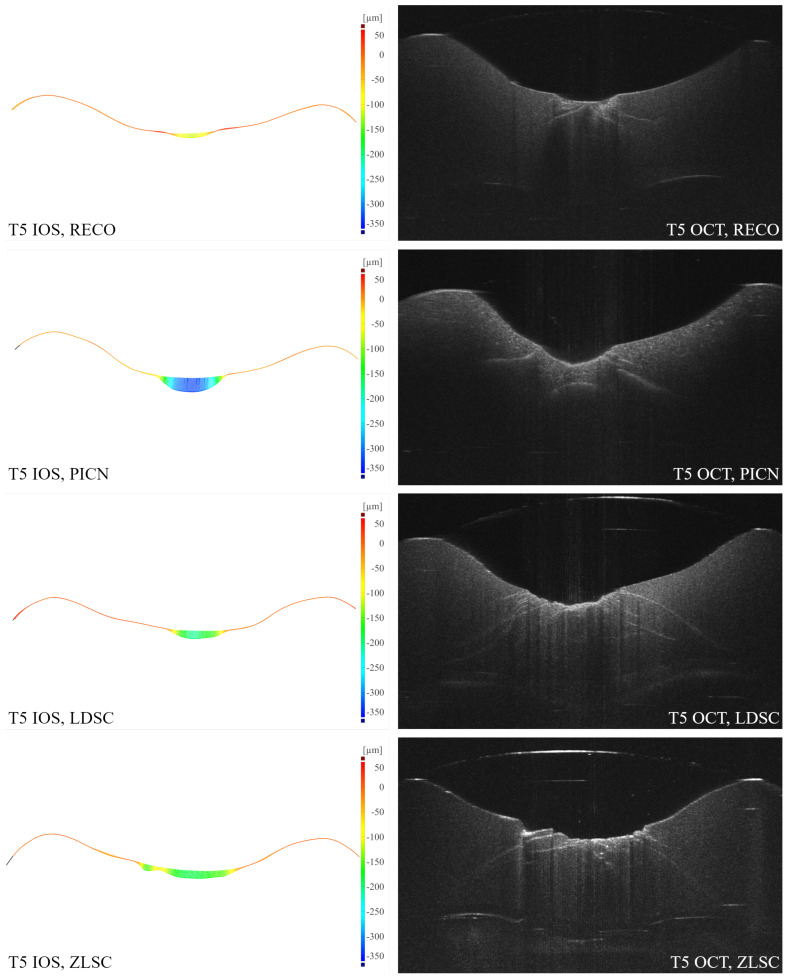
Comparison of surface wear and subsurface damage at T5 (after 1,250,000 load cycles) across all material classes; resin composite (RECO), polymer-infiltrated ceramic network (PICN), lithium disilicate ceramic (LDSC), and zirconia-reinforced lithium silicate ceramic (ZLSC). **Left**: Intraoral scanning (IOS) surface deviation maps referenced to T0. **Right**: Representative optical coherence tomography (OCT) cross-sections illustrating the qualitative extent and propagation of subsurface damage. Due to optical distortion caused by the water droplet used to reduce specular reflection and improve image contrast, the OCT images are intended for qualitative visualization only and do not provide reliable absolute spatial dimensions. RECO data previously reported in Kuhl et al. [1].

Despite the high failure rate in ZLSC, fatigue damage was detectable by both modalities from the earliest timepoint in all material classes. Both IOS and OCT revealed progressive damage over time across all four material classes, with OCT consistently indicating higher defect extents than IOS. Figure 2 shows a representative comparison of IOS-derived surface deviation maps and OCT-derived cross-sectional images at T5 for each material class. The images represent a longitudinal cross-section through the crown along the occlusal contact area; for each material class, the left image shows the IOS surface deviation map referenced to baseline (T0) and the right image shows the corresponding OCT cross-section. While both modalities capture progressive damage, their patterns differ fundamentally: IOS reflects external surface wear, whereas OCT reveals subsurface damage propagation, illustrating the systematic discrepancy between the two modalities across material classes.

### 3.2. Vertical Defect Propagation

**Figure 3:**
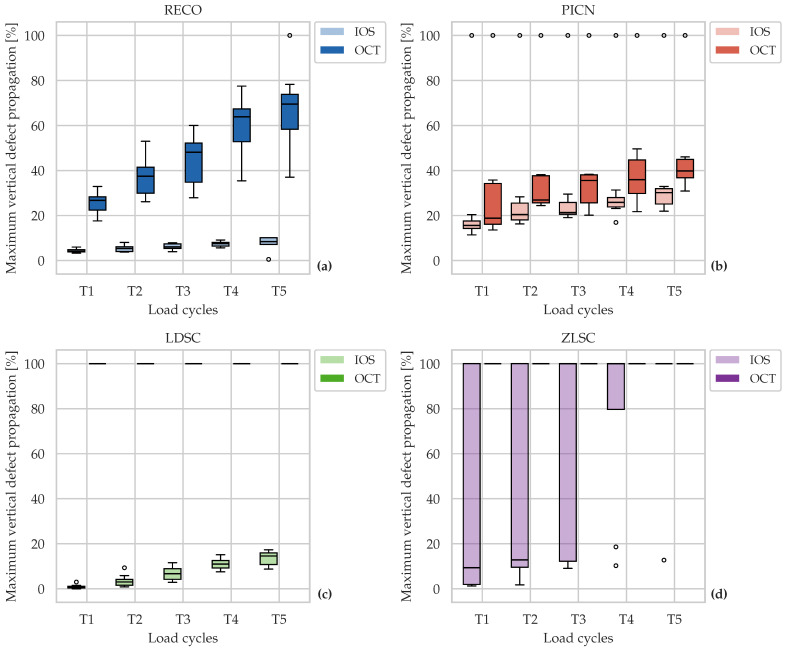
Maximum vertical defect propagation (%) by intraoral scanning (IOS, surface wear) and optical coherence tomography (OCT, subsurface damage) for four CAD/CAM crown materials: (**a**) resin composite (RECO, Brilliant Crios), (**b**) polymer-infiltrated ceramic network (PICN, Vita Enamic), (**c**) lithium disilicate ceramic (LDSC, IPS e.max CAD), and (**d**) zirconia-reinforced lithium silicate ceramic (ZLSC, Cerec Tessera). OCT values are normalized to local occlusal thickness and IOS values to a 1.5 mm reference. RECO data were previously reported in Kuhl et al. [1] and are included for cross-material comparison. Collapsed boxes (single horizontal line at 100%) indicate that all specimens within that group reached the maximum value, defined as either full-thickness cracking through the entire crown layer or catastrophic failure of the crown. Timepoints: after 250,000 (T1), 500,000 (T2), 750,000 (T3), 1,000,000 (T4), and 1,250,000 (T5) load cycles.

Figure 3 displays the maximum vertical defect propagation for all material classes across T1–T5. All four material classes showed progressive damage over time in both modalities, with OCT yielding systematically higher values than IOS throughout. For RECO and the PICN, both IOS and OCT revealed a monotonic increase in central tendency, consistent with gradual fatigue damage accumulation. For LDSC and ZLSC, OCT vertical measurements reached the maximum value of 100% from T1 onward, indicating full-thickness vertical crack propagation from the earliest observation interval; as a result, the OCT boxplots for these materials collapse to a single horizontal line at 100%, reflecting the absence of variance across specimens. IOS-derived surface wear remained substantially lower for LDSC across all timepoints. For ZLSC, the progressive specimen loss resulted in an increasing proportion of 100% values in the IOS data as well as fractured specimens were assigned maximum damage values; this is reflected in the wide IQR and the upper extent of the IOS boxplots approaching 100% at later timepoints. The dissociation between externally visible surface wear and subsurface crack depth was most pronounced in LDSC and ZLSC and reflects the capacity of OCT to detect subsurface damage that does not manifest proportionally at the surface. Detailed descriptive statistics are provided in Table A1.

### 3.3. Horizontal Defect Propagation

**Figure 4:**
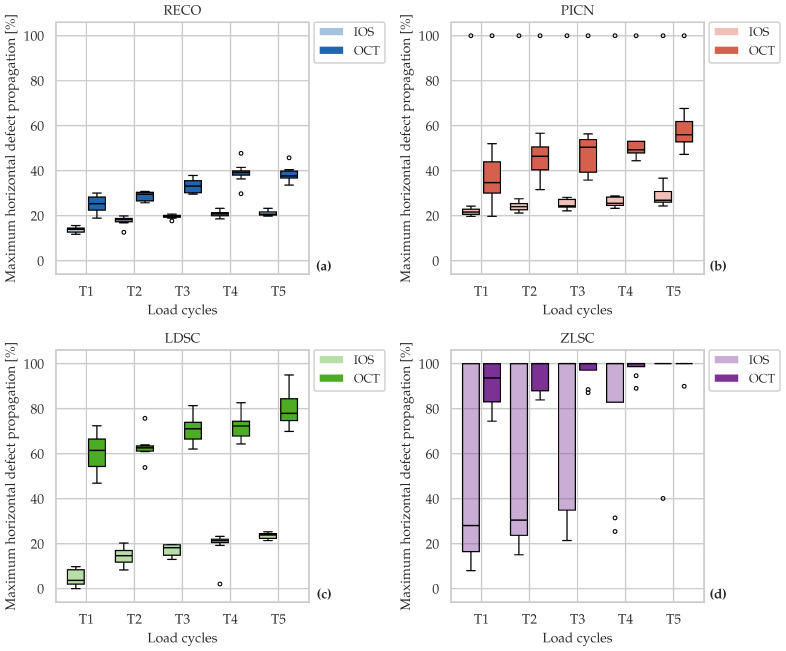
Maximum horizontal defect propagation (%) by intraoral scanning (IOS, surface wear) and optical coherence tomography (OCT, subsurface damage) for four CAD/CAM crown materials: (**a**) resin composite (RECO, Brilliant Crios), (**b**) polymer-infiltrated ceramic network (PICN, Vita Enamic), (**c**) lithium disilicate ceramic (LDSC, IPS e.max CAD), and (**d**) zirconia-reinforced lithium silicate ceramic (ZLSC, Cerec Tessera). OCT values are normalized to the cusp-tip distance; IOS values to a 7 mm reference. RECO data were previously reported in Kuhl et al. [1] and are included for cross-material comparison. Collapsed boxes (single horizontal line at 100%) indicate that all specimens within that group reached the maximum value, defined as either full-thickness cracking through the entire crown layer or catastrophic failure of the crown. Timepoints: after 250,000 (T1), 500,000 (T2), 750,000 (T3), 1,000,000 (T4), and 1,250,000 (T5) load cycles.

Figure 4 displays the maximum horizontal defect propagation for all material classes across T1–T5. Consistent with the vertical metrics, OCT yielded higher values than IOS across all material classes and timepoints, and both modalities showed progressive increases over time. For the PICN and ZLSC, considerable variability was observed, particularly in the IOS data, driven in part by the 100% values assigned to fractured specimens. For ZLSC, OCT horizontal values were already markedly elevated from T1, though without the complete ceiling effect seen in the vertical metric, as evidenced by the remaining variance in the OCT boxplots at early timepoints. Notably, between-method differences were smaller for the horizontal than for the vertical metric across all material classes, suggesting that horizontal crack propagation is more closely reflected by external surface changes than vertical crack depth. Detailed descriptive statistics are provided in Table A2.

### 3.4. Correspondence Between IOS and OCT

**Figure 5:**
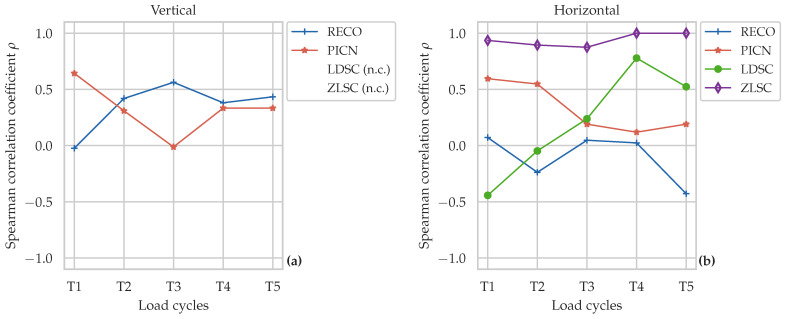
Spearman’s rank correlation coefficient (ρ) between IOS-derived surface wear and OCT-derived subsurface damage for vertical (**a**) and horizontal (**b**) metrics for four CAD/CAM crown materials: resin composite (RECO, Brilliant Crios), polymer-infiltrated ceramic network (PICN, Vita Enamic), lithium disilicate ceramic (LDSC, IPS e.max CAD), and zirconia-reinforced lithium silicate ceramic (ZLSC, Cerec Tessera). In panel (**a**), ρ could not be calculated for LDSC and ZLSC (n.c.) as OCT vertical values reached a ceiling of 100% at all timepoints, reflecting predominantly vertical crack propagation in these materials. RECO data were previously reported in Kuhl et al. [1] and are included for cross-material comparison. Timepoints: after 250,000 (T1), 500,000 (T2), 750,000 (T3), 1,000,000 (T4), and 1,250,000 (T5) load cycles.

Spearman’s rank correlation coefficients between IOS- and OCT-derived metrics are displayed in Figure 5 and reported in full in Table A3 and Table A4.

Regarding the vertical metric, ρ could not be calculated for LDSC and ZLSC due to the OCT ceiling effect described above. Neither RECO nor the PICN showed statistically significant associations at any timepoint (all p>0.05). Across assessment timepoints, effect sizes following the conventions of Cohen [38] varied from small negative (|ρ|<0.10) to medium positive values (|ρ| up to 0.563 for RECO and 0.643 for the PICN), yet all tests remained non-significant, providing no evidence of a reliable association between vertical surface wear and subsurface crack depth for these materials. Consistent with the small sample size, the corresponding 95% bootstrap confidence intervals were wide and included zero throughout (Table A3), underscoring the limited precision of these estimates.

Horizontal correlations showed a more differentiated picture across material classes. RECO and the PICN yielded no significant associations at any timepoint. In LDSC, a single significant correlation emerged at T4 (ρ=0.778, p=0.023), while all other timepoints remained non-significant; however, its 95% confidence interval was wide and included zero (95% CI: −0.190 to 1.000), indicating that this isolated association is statistically fragile and should not be over-interpreted. ZLSC showed consistently strong and statistically significant correlations across all horizontal timepoints (ρ=0.875–1.000, all p≤0.004). However, these values must be interpreted with caution: the high ρ values at T4 and T5 in particular are likely driven by the progressive specimen loss in ZLSC, where the majority of fractured specimens were assigned 100% for both IOS and OCT, producing near-identical ranks across modalities rather than reflecting a true biological association between surface wear and subsurface damage. This interpretation is reinforced by the confidence intervals, which collapsed to a zero-width interval at T4 and T5 (95% CI: 1.000 to 1.000) as a direct consequence of the identical 100% ranks assigned to fractured specimens, rather than reflecting a genuine association.

Taken together, these findings provide no consistent evidence of a reliable association between IOS-derived surface wear and OCT-derived subsurface damage propagation across the tested material classes. Accordingly, the null hypothesis that surface wear measured by IOS is not associated with subsurface damage propagation measured by OCT could not be rejected for RECO and the PICN. For LDSC and ZLSC, the null hypothesis could not be formally tested for the vertical metric due to the OCT ceiling effect, and the significant horizontal correlations observed for ZLSC are likely confounded by specimen failure as described above.

## 4. Discussion

This multi-material in vitro study extended the dual-modality monitoring workflow established in Kuhl et al. [1] to three additional CAD/CAM crown materials representing distinct material classes. Under clinical-close phantom-head conditions, both IOS and OCT revealed progressive fatigue damage across all four material classes, yet the correspondence between the two modalities differed substantially depending on material-specific fracture behavior. Externally visible surface wear assessed by IOS did not reliably reflect subsurface damage propagation detected by OCT in any of the tested material classes, extending the findings of Kuhl et al. [1] beyond resin composites to a broader range of CAD/CAM material classes.

RECO data from Kuhl et al. [1] were included to enable direct cross-material comparison under identical conditions. The present study was intentionally designed as a direct continuation of the preceding single-material investigation, with specimen preparation, loading parameters, and monitoring procedures held constant across both studies; only the crown material and the material-specific adhesive luting protocols differed. This design minimizes potential confounding effects arising from protocol variations and enables the RECO data to serve as a valid reference for cross-material comparisons.

A general mechanical principle relevant to all four material classes concerns the stress distribution under Hertzian contact loading: compressive stresses dominate directly beneath the antagonist, while tensile stresses develop at the contact periphery [17]. Since dental restorative materials fail preferentially under tension [12,14,17], and exhibit a marked tension-compression asymmetry [11], crack initiation occurs preferentially at the contact periphery, favoring horizontally oriented propagation. In more brittle silicate-based materials, this peripheral tensile initiation is additionally accompanied by rapid vertical full-thickness propagation, as visible in the OCT cross-sections of LDSC and ZLSC in Figure 2. This damage morphology also explains why horizontal propagation was more closely mirrored by external surface changes than vertical propagation across all material classes. The OCT cross-sections (Figure 2) were dominated by cone cracks (outer, inner, and median) initiating at the periphery of the loaded contact, most pronounced at the crater rim in LDSC and ZLSC and more central in RECO and the PICN. Consistent with the purely axial loading, neither partial cone cracks nor radial or flexure cracks from the cementation side [12] were observed. Because the near-surface, horizontally oriented segment of a cone crack coincides with the region of occlusal material loss, its lateral extension is partially captured by IOS, whereas the vertical cone-crack penetration advances beneath an essentially intact surface—accounting for the closer IOS-OCT correspondence in the horizontal metric across all material classes.

For RECO and the PICN, both modalities revealed progressive fatigue damage over time, yet no statistically significant association between surface wear and subsurface damage propagation was observed at any timepoint for either material class (RECO: p=0.146–0.955; PICN: p=0.086–0.978). Compared to RECO, the PICN showed a smaller IOS-OCT discrepancy in the vertical metric, while horizontal OCT values and the corresponding IOS-OCT difference were both larger than observed for RECO. The pronounced occlusal abrasion of the PICN [3] substantially raises IOS-derived vertical surface loss values, thereby narrowing the relative IOS-OCT vertical discrepancy. Simultaneously, the progressive formation of a broad wear facet—visible in Figure 2—transforms the initial point contact into a more distributed load geometry, further promoting horizontal crack propagation as outlined above. This behavior reflects the material properties of the PICN: with an elastic modulus of ~30 GPa—placing it between enamel and dentin and roughly threefold above that of resin-based composites—and a flexural strength of ~150 MPa it tends toward brittle, ceramic-like behavior [7,8,9], in contrast to RECO, which exhibits a lower stiffness and a higher plastic deformation component that absorbs cyclic loading energy, reducing surface material loss while subsurface fatigue damage accumulates [11]. One PICN specimen fractured between T0 and T1, while all remaining PICN specimens survived the complete observation period—an isolated event that is difficult to attribute solely to material behavior under the applied loading conditions. It cannot be excluded that a pre-existing material defect, a luting irregularity, or an undetected preparation artifact contributed to this event; it should therefore be interpreted with caution rather than as representative of PICN fatigue behavior under the present conditions.

The most striking finding of the present study was the immediate and complete vertical subsurface damage propagation detected by OCT in LDSC and ZLSC from T1 onward. All surviving specimens reached 100% vertical defect propagation at the earliest observation interval, precluding Spearman correlation analysis for the vertical metric in both material classes. This ceiling effect reflects the rapid vertical full-thickness crack propagation outlined above and is consistent with the brittle fracture behavior of silicate-based ceramics under cyclic occlusal loading documented in prior fatigue studies [3,12,15,17]. The clinical implication is substantial: full-thickness vertical subsurface damage propagation was present after only 250,000 load cycles—equivalent to approximately one year of simulated clinical function [35]—while IOS-derived surface wear remained markedly lower, particularly for LDSC. This pronounced dissociation suggests that IOS-based monitoring alone would fail to detect clinically consequential subsurface damage at an early and potentially still treatable stage in these material classes, directly addressing the limitation identified in Kuhl et al. [1]. In the horizontal dimension, no ceiling effect was observed for LDSC, where OCT-derived horizontal damage propagation was substantially elevated throughout but increased progressively; no significant IOS-OCT association was found at most timepoints, with the exception of T4 (ρ=0.778, p=0.023). For ZLSC, horizontal OCT values were markedly elevated from T1, and the consistently high Spearman correlations (ρ=0.875–1.000, all p≤0.004) are likely a statistical artifact of the progressive specimen loss rather than a genuine IOS-OCT association. It should be noted that the present worst-case loading protocol—single-point contact at 500 N over 1,250,000 cycles—represents a particularly severe fatigue scenario for brittle silicate-based ceramics, which exhibit substantially lower damage tolerance under concentrated cyclic contact loading than resin-based materials [12,17,19,39]. In line with this, chairside-generated monolithic LDSC crowns have shown favorable medium-term clinical survival, with a reported failure-free rate of 87.6% after six years [40]. A comparable picture emerges for ZLSC: in a prospective practice-based study, chairside-fabricated monolithic ZLSC partial crowns (Celtra Duo, Dentsply Sirona) achieved a 99% survival and 98% success rate after three years, with only a single material fracture among 88 restorations [41]. Accordingly, the early full-thickness subsurface crack propagation observed here under concentrated worst-case loading conditions should not be equated with clinical failure for physiological occlusal function. Notably, the ZLSC material investigated here (Cerec Tessera, Dentsply Sirona) differs from the ZLSC material used by Schlenz et al. [3] (Celtra Duo, Dentsply Sirona), and the markedly higher catastrophic failure rate observed here—seven of eight specimens—compared to the absence of failures reported in that study may partly reflect compositional differences between these products in addition to the extended loading duration (1,250,000 vs. 1,000,000 cycles). Future studies should investigate silicate-based materials under loading conditions more closely aligned with their clinical indications and with larger sample sizes to compensate for specimen loss.

To the best of the authors’ knowledge, only one prior study has monitored fatigue damage in multiple CAD/CAM materials using OCT [3], while two single-material investigations exist: one for lithium disilicate veneers [29] and one for CAD/CAM resin composite using the identical dual-modality IOS-OCT workflow of the present study [1]. Yazigi et al. [29] detected subsurface cracks in 23% of specimens after thermo-mechanical loading, with pre-existing cracks associated with more catastrophic failure—directly supporting the clinical relevance of subsurface damage assessment independent of surface appearance. Schlenz et al. [3] found progressive subsurface damage across RECO, PICN and ZLSC materials with ZLSC showing the most pronounced defects; all materials except the high-strength zirconia groups already exhibited crack formation after 250,000 cycles—consistent with the early OCT-detected damage observed across all material classes in the present study. Yazigi et al. [29] and Schlenz et al. [3] employed non-handheld laboratory OCT systems unsuitable for intraoral use and did not directly compare subsurface damage propagation with surface wear, while Kuhl et al. [1] established the identical handheld IOS-OCT workflow but restricted the comparison to a single material class. The present study builds on these findings by extending the chairside-compatible dual-modality workflow to three additional CAD/CAM material classes, enabling a direct cross-material comparison of IOS-OCT correspondence under identical conditions.

Taken together, these findings have direct clinical implications: the IOS-OCT discrepancy observed across all material classes reinforces the conclusion that surface-based monitoring alone is insufficient to reflect subsurface fatigue damage progression regardless of material class—most strikingly for LDSC and ZLSC, where OCT detected full-thickness vertical subsurface damage at T1 while IOS showed only minimal surface wear. In a clinical scenario, standard recall-based surface monitoring would not raise concern at this stage, while subsurface OCT imaging would already indicate advanced subsurface damage. Early detection matters: subsurface cracks that have not yet propagated through the full restoration thickness may still permit minimally invasive repair [12,29], whereas full-thickness vertical crack propagation—as detected from T1 onward in LDSC and ZLSC—likely necessitates restoration replacement. Intraoral OCT may thus enable timely intervention before irreversible failure occurs, supporting individualized recall scheduling and repair decisions [1]; this added value appears particularly pronounced for silicate-based materials, where the dissociation between surface wear and subsurface damage is greatest.

The following limitations must be acknowledged. The in vitro phantom-head model cannot fully replicate the biological and mechanical complexity of the oral environment, including patient-specific factors such as saliva, biofilm, head movement, and thermal fluctuations; clinical studies are necessary to validate feasibility and predictive accuracy in vivo [1]. The sample size was limited (n=8 per material), and no a priori power calculation was performed; findings are therefore exploratory and should be interpreted with caution. The high failure rate in ZLSC substantially reduced the effective sample size at later timepoints (down to *n* = 1 at T5), which markedly limits the reliability and generalizability of the statistical analyses for this material; the corresponding late-timepoint results should therefore be regarded as descriptive rather than confirmatory. Restricting the analysis to surviving specimens would not remedy this: with only a single survivor at T5, no meaningful statistic or boxplot can be derived, and excluding fractured specimens would misrepresent the actual damage state by disregarding complete failures. Future studies should consider larger sample sizes and adapted loading protocols for silicate-based material classes. Fractured specimens were assigned 100% for both modalities from the timepoint of fracture onward, representing a conservative worst-case imputation; while methodologically justified, this approach influenced descriptive statistics and Spearman correlations for ZLSC and should be considered when interpreting these results. OCT imaging required a distilled-water droplet to optimize image contrast, introducing case-specific lensing that precluded the use of absolute metric values; geometry-normalized relative percentages were therefore reported, as described in detail in Kuhl et al. [1]. All measurements were performed by a single non-blinded examiner; despite a standardized protocol, a potential measurement bias—particularly for manual defect-extent identification on OCT cross-sections—cannot be excluded, and future studies should incorporate blinded assessment. Finally, all material classes were tested under identical worst-case loading conditions; material-specific loading protocols adjusted to clinical indications may yield different results and warrant investigation in future studies.

## 5. Conclusions

Within the limitations of this exploratory multi-material in vitro study, dual-modality monitoring under clinical-close conditions revealed progressive surface wear and subsurface damage propagation across all four CAD/CAM crown material classes. Externally visible surface wear assessed by IOS did not reliably reflect subsurface damage propagation detected by OCT at any assessment timepoint, regardless of material class, extending the findings of a previous single-material investigation to a broader range of restorative materials. The dissociation between the two modalities was most pronounced in silicate-based materials, where OCT detected full-thickness vertical subsurface damage propagation from the earliest timepoint while IOS-derived surface wear remained markedly lower. Surface-based assessment alone may therefore substantially underestimate clinically relevant internal fatigue damage. Accordingly, complementary OCT imaging provided the least additional diagnostic value for the more damage-tolerant RECO and PICN, for which surface-based recall inspection may be more acceptable, and the most for the brittle silicate-based materials LDSC and ZLSC, which may benefit most from subsurface-sensitive monitoring. Intraoral OCT may complement routine IOS by providing non-invasive, chairside information on subsurface damage propagation and may thereby support individualized recall scheduling and minimally invasive repair decisions. However, its added value for fatigue monitoring needs to be confirmed in larger in vitro and clinical studies across a broader range of loading conditions and restoration designs.

## Figures and Tables

**Table 1:** Materials used in the study (information provided by manufacturer).

Code	Material Class	Product Name (Batch No.)	Polishing System (Batch No.)	Pre- Treatment	Bonding Agent (Batch No.)	Luting System (Batch No.)
RECO	Resin composite	Brilliant Crios ^a^ (L67588)	DIATECH Finishing & Polishing Kit ^a^ (305800)	Air abrasion, 50 μm Al_2_O_3_, 1.5 bar	One Coat 7 Universal ^a^ (L64740)	DuoCem ^a^ (L63105)
PICN	Polymer- infiltrated ceramic network	Vita Enamic ^b^ (94890)	Vita Enamic Polishing Set technical ^b^ (E76470)	5% HF, IPS Ceramic Ethcing Gel ^c^, 60 s	Vita Adiva C-Prime ^b^ (89940), Vita Adiva T-Bond I/II ^b^ (91900)	Vita Adiva F-Cem ^b^ (97930)
LDSC	Lithium disilicate ceramic	IPS e.max CAD ^c^ (Z034M8)	OptraFine F ^c^ (VL0798), OptraFine P ^c^ (VL0760)	5% HF, IPS Ceramic Ethcing Gel ^c^, 20 s	Clearfil Ceramic Primer Plus ^d^ (230073), ED Primer II ^d^ (A: 7T0060, B: 2M0062)	Panavia F2.0 ^d^ (A: 2N0265, B: 8S0113)
ZLSC	Zirconia- reinforced lithium silicate ceramic	Cerec Tessera ^e^ (16013757)	Renfert all-in-one ^f^ (n/a)	5% HF, IPS Ceramic Ethcing Gel ^c^, 60 s	Prime&Bond active ^e^ (2112000640)	Calibra Ceram ^e^ (00098215)

^a^ Coltene, Altstätten, Switzerland; ^b^ VITA Zahnfabrik, Bad Säckingen, Germany; ^c^ Ivoclar Vivadent, Schaan, Liechtenstein; ^d^ Kuraray, Okayama, Japan; ^e^ Dentsply Sirona, Bensheim, Germany; ^f^ Renfert, Hilzingen, Germany. HF = hydrofluoric acid; Al_2_O_3_ = aluminum oxide; n/a = not available.

## Data Availability

The datasets in this article are available from the corresponding author upon reasonable request.

## Abbreviations

The following abbreviations are used in this manuscript:

CAD/CAM	Computer-aided design/computer-aided manufacturing
CNC	Computer numerical control
IOS	Intraoral scanning
IQR	Interquartile range
LDSC	Lithium disilicate ceramic
OCT	Optical coherence tomography
PICN	Polymer-infiltrated ceramic network
RECO	Resin composite
SD	Standard deviation
SS-OCT	Swept-source optical coherence tomography
ZLSC	Zirconia-reinforced lithium silicate ceramic

## Appendix A

**Table A1:** Descriptive statistics for IOS- and OCT-derived vertical defect propagation (%) at each timepoint, reported as mean ± SD and median (IQR). RECO data previously reported in Kuhl et al. [1].

Material Class	Method	T1	T2	T3	T4	T5
RECO	IOS	4.36±0.91	5.33±1.47	6.18±1.36	7.33±1.22	7.78±3.19
		4.13 (1.03)	5.33 (2.12)	6.07 (2.07)	7.50 (1.63)	8.43 (2.93)
	OCT	25.47±4.97	37.15±8.88	44.67±12.17	59.44±13.99	66.79±19.53
		26.73 (5.88)	37.45 (11.54)	48.14 (17.41)	63.86 (14.54)	69.52 (15.47)
PICN	IOS	25.99±30.01	30.68±28.30	32.03±27.67	34.25±26.87	36.94±25.80
		15.63 (3.25)	20.43 (7.50)	21.30 (5.22)	25.83 (4.15)	30.17 (6.82)
	OCT	31.52±28.93	38.08±25.62	39.73±25.33	43.01±24.65	46.50±22.23
		18.84 (18.15)	26.92 (12.02)	35.57 (12.40)	35.95 (14.90)	39.81 (8.18)
LDSC	IOS	0.93±0.97	3.60±2.80	6.71±3.13	11.00±2.56	13.57±3.34
		0.63 (0.75)	3.03 (2.60)	6.70 (4.72)	10.97 (3.32)	14.57 (5.15)
	OCT	100.00±0.00	100.00±0.00	100.00±0.00	100.00±0.00	100.00±0.00
		100.00 (0.00)	100.00 (0.00)	100.00 (0.00)	100.00 (0.00)	100.00 (0.00)
ZLSC	IOS	40.46±49.44	43.08±47.28	66.45±46.32	78.61±39.67	89.09±30.85
		9.37 (98.05)	12.87 (90.40)	100.00 (87.80)	100.00 (20.35)	100.00 (0.00)
	OCT	100.00±0.00	100.00±0.00	100.00±0.00	100.00±0.00	100.00±0.00
		100.00 (0.00)	100.00 (0.00)	100.00 (0.00)	100.00 (0.00)	100.00 (0.00)

Values reported as mean ± SD (top row) and median (IQR) (bottom row). Timepoints: after 250,000 (T1), 500,000 (T2), 750,000 (T3), 1,000,000 (T4), and 1,250,000 (T5) load cycles. RECO = resin composite; PICN = polymer-infiltrated ceramic network; LDSC = lithium disilicate ceramic; ZLSC = zirconia-reinforced lithium silicate ceramic; IOS = intraoral scanning; OCT = optical coherence tomography; SD = standard deviation; IQR = interquartile range.

**Table A2:** Descriptive statistics for IOS- and OCT-derived horizontal defect propagation (%) at each timepoint, reported as mean ± SD and median (IQR). RECO data previously reported in Kuhl et al. [1].

Material Class	Method	T1	T2	T3	T4	T5
RECO	IOS	13.71±1.28	17.68±2.29	19.63±0.97	20.71±1.44	21.06±1.18
		13.92 (1.78)	18.21 (1.44)	19.94 (0.82)	20.71 (1.35)	20.68 (1.36)
	OCT	25.19±3.99	28.66±2.07	33.14±3.24	38.95±4.99	38.42±3.67
		25.30 (5.76)	29.54 (3.78)	33.12 (5.36)	39.16 (1.87)	37.70 (3.13)
PICN	IOS	31.29±27.80	33.29±27.02	34.15±26.68	35.02±26.32	36.90±25.78
		21.59 (2.24)	24.00 (2.76)	24.34 (3.41)	25.47 (3.73)	26.89 (4.74)
	OCT	42.52±25.18	50.89±21.31	52.86±20.55	55.58±18.18	61.29±16.87
		34.70 (13.85)	46.36 (10.15)	50.42 (14.50)	49.33 (5.14)	55.95 (9.01)
LDSC	IOS	4.60±3.88	14.49±4.14	17.12±2.79	19.04±6.97	23.47±1.37
		3.67 (6.34)	14.68 (5.16)	18.21 (4.64)	21.36 (1.52)	23.85 (2.06)
	OCT	60.18±9.61	63.00±6.03	70.92±6.13	71.95±5.66	79.79±8.21
		61.45 (12.20)	62.57 (2.29)	71.06 (7.46)	72.31 (6.61)	77.93 (9.71)
ZLSC	IOS	49.30±42.63	52.42±39.82	73.24±37.19	82.11±33.17	92.51±21.18
		28.06 (83.52)	30.47 (76.31)	100.00 (65.09)	100.00 (17.14)	100.00 (0.00)
	OCT	90.55±10.83	94.68±7.51	96.95±5.65	97.96±4.06	98.75±3.55
		93.66 (16.97)	100.00 (12.05)	100.00 (2.87)	100.00 (1.34)	100.00 (0.00)

Values reported as mean ± SD (top row) and median (IQR) (bottom row). Timepoints: after 250,000 (T1), 500,000 (T2), 750,000 (T3), 1,000,000 (T4), and 1,250,000 (T5) load cycles. RECO = resin composite; PICN = polymer-infiltrated ceramic network; LDSC = lithium disilicate ceramic; ZLSC = zirconia-reinforced lithium silicate ceramic; IOS = intraoral scanning; OCT = optical coherence tomography; SD = standard deviation; IQR = interquartile range.

**Table A3:** Spearman’s rank correlation coefficient (ρ), 95% confidence interval (CI) and *p*-value between IOS-derived surface wear and OCT-derived subsurface damage for the vertical metric at each timepoint. RECO data previously reported in Kuhl et al. [1].

Material Class	Statistic	T1	T2	T3	T4	T5
RECO	ρ	−0.024	0.419	0.563	0.381	0.434
	95% CI	[−0.788,0.679]	[−0.519,0.918]	[−0.192,0.955]	[−0.707,1.000]	[−0.477,0.981]
	*p*	0.955	0.301	0.146	0.352	0.283
PICN	ρ	0.643	0.310	−0.012	0.333	0.333
	95% CI	[−0.235,1.000]	[−0.671,1.000]	[−0.961,0.900]	[−0.671,1.000]	[−0.671,1.000]
	*p*	0.086	0.456	0.978	0.420	0.420
LDSC	ρ	n.c.	n.c.	n.c.	n.c.	n.c.
	95% CI	n.c.	n.c.	n.c.	n.c.	n.c.
	*p*	n.c.	n.c.	n.c.	n.c.	n.c.
ZLSC	ρ	n.c.	n.c.	n.c.	n.c.	n.c.
	95% CI	n.c.	n.c.	n.c.	n.c.	n.c.
	*p*	n.c.	n.c.	n.c.	n.c.	n.c.

Two-sided tests, α=0.05; no adjustment for multiple comparisons. n.c. = not calculable; OCT vertical values were at ceiling (100%) across all timepoints for LDSC and ZLSC, precluding rank variation. Timepoints: after 250,000 (T1), 500,000 (T2), 750,000 (T3), 1,000,000 (T4), and 1,250,000 (T5) load cycles. RECO = resin composite; PICN = polymer-infiltrated ceramic network; LDSC = lithium disilicate ceramic; ZLSC = zirconia-reinforced lithium silicate ceramic.

**Table A4:** Spearman’s rank correlation coefficient (ρ), 95% confidence interval (CI) and *p*-value between IOS-derived surface wear and OCT-derived subsurface damage for the horizontal metric at each timepoint. RECO data previously reported in Kuhl et al. [1].

Material Class	Statistic	T1	T2	T3	T4	T5
RECO	ρ	0.071	−0.238	0.048	0.024	−0.429
	95% CI	[−0.684,0.795]	[−0.926,0.615]	[−0.846,0.753]	[−0.778,0.679]	[−0.926,0.468]
	*p*	0.867	0.570	0.911	0.955	0.289
PICN	ρ	0.595	0.548	0.190	0.119	0.190
	95% CI	[−0.205,1.000]	[−0.259,1.000]	[−0.737,0.923]	[−0.848,0.852]	[−0.620,1.000]
	*p*	0.120	0.160	0.651	0.779	0.651
LDSC	ρ	−0.443	−0.048	0.238	0.778 *	0.524
	95% CI	[−0.926,0.342]	[−0.823,0.753]	[−0.620,0.852]	[−0.190,1.000]	[−0.235,0.852]
	*p*	0.272	0.911	0.570	0.023	0.183
ZLSC	ρ	0.936 *	0.894 *	0.875 *	1.000 *	1.000 *
	95% CI	[0.632,1.000]	[0.619,1.000]	[0.632,1.000]	[1.000,1.000]	[1.000,1.000]
	*p*	0.001	0.003	0.004	<0.001	<0.001

Two-sided tests, α=0.05; no adjustment for multiple comparisons. * p<0.05. Timepoints: after 250,000 (T1), 500,000 (T2), 750,000 (T3), 1,000,000 (T4), and 1,250,000 (T5) load cycles. RECO = resin composite; PICN = polymer-infiltrated ceramic network; LDSC = lithium disilicate ceramic; ZLSC = zirconia-reinforced lithium silicate ceramic.
